# Heart rate to identify non-febrile children with dehydration and acute kidney injury in emergency department: a prospective validation study

**DOI:** 10.1007/s00431-024-05770-6

**Published:** 2024-09-16

**Authors:** Pierluigi Marzuillo, Giulio Rivetti, Antonietta Galeone, Giusy Capasso, Paola Tirelli, Anna Di Sessa, Emanuele Miraglia del Giudice, Stefano Guarino, Felice Nunziata

**Affiliations:** 1 Department of Woman, Child and of General and Specialized Surgery, Università Degli Studi Della Campania “Luigi Vanvitelli”, Via Luigi De Crecchio 2, 80138 Naples, Italy; 2 Department of Pediatrics, AORN Sant’Anna E San Sebastiano, Via Ferdinando Palasciano, 81100 Caserta, Italy

**Keywords:** Acute kidney injury, Heart rate, Dehydration, Children

## Abstract

We previously developed and retrospectively validated the estimated percentage of heart rate variation (EHRV) as a predictor of the composite outcome of ≥ 5% dehydration and/or acute kidney injury (AKI) in non-febrile children. The current study aimed to prospectively validate EHRV as a predictor for dehydration or AKI in a different cohort of children attending the Pediatric Emergency Department. From July 2022 to August 2023, 256 pediatric patients aged 0–18 years attending the Pediatric Emergency Department were enrolled. EHRV was calculated as follows: [(HR at admission** − **50th percentile of HR for age and sex)/HR at admission] × 100. Dehydration was categorized as < 5% or ≥ 5% fluid deficit. AKI was defined according to KDIGO creatinine criteria. Statistical analyses included receiver-operating characteristic (ROC) curves and logistic regression analysis. Among enrolled patients, 52 had ≥ 5% dehydration, 50 had AKI, and 16 had both conditions. EHRV demonstrated significant predictive ability for both ≥ 5% dehydration (AUROC = 0.71; 95% confidence interval (CI), 0.63–0.78; *p* < 0.001) and AKI (AUROC = 0.78; 95% CI, 0.71–0.84; *p* < 0.001). An EHRV > 24.5% was associated with an increased odds ratio (OR), adjusted for confounders, of ≥ 5% dehydration (OR = 3.5; 95% CI, 1.6–8.0; *p* = 0.003) and AKI (OR = 3.4; 95% CI, 1.6–7.3; *p* = 0.002). The sensitivity and specificity of this cut-off were 34% and 83% for ≥ 5% dehydration and 36% and 84% for AKI, respectively.

*Conclusions*: This study prospectively validates the clinical utility of EHRV in predicting dehydration and AKI in a pediatric emergency care setting. An EHRV > 24.5% could serve as a marker for suspecting dehydration or AKI. Further validation across diverse patient populations and settings is needed.

**Table:** 

**What is Known:** • *An increased heart rate (HR) is a readily detectable sign of dehydration in children.* • *In a retrospective validation cohort, an estimated HR variation (EHRV) greater than 24.5% compared to the 50th percentile of HR was predictive of* ≥ *5% dehydration and/or acute kidney injury (AKI) in non-febrile patients.*
**What is New:** • *We prospectively validated the clinical utility of EHRV in predicting dehydration and AKI in a pediatric emergency care setting.* • *We confirmed that an EHRV greater than 24.5% is associated with increased odds of* ≥ *5% dehydration and AKI.*

**Supplementary Information:**

The online version contains supplementary material available at 10.1007/s00431-024-05770-6.

## Introduction

Dehydration is a condition characterized by the excessive loss of body water, with vomiting and diarrhea being the most common causes in children [1].

Early identification of dehydration symptoms and accurate assessment are crucial to prevent complications [1]. As dehydration progresses, the body compensates by increasing heart rate (HR) to maintain cardiac output and peripheral perfusion despite reduced blood volume [2]. Dehydration can also decrease renal perfusion, potentially leading to acute kidney injury (AKI) [3–5]. Therefore, an elevated HR may indirectly indicate compromised renal perfusion, warranting further investigation into the underlying cause [6].

Although an elevated HR is commonly recognized as a sign of dehydration in children due to its ease of detection, definitive cut-off values for HR that accurately define dehydration are currently lacking [1].

In a previous study, we developed and retrospectively validated the estimated percentage of heart rate variation (EHRV) in acute setting as a predictor of the composite outcome of ≥ 5% dehydration and/or AKI [7]. The optimal EHRV cut-off for predicting both AKI and dehydration was 24.5% [7]. The aim of this study was to prospectively validate the previously identified EHRV cut-off for diagnosing ≥ 5% dehydration or AKI in children presenting to the Pediatric Emergency Department.

## Methods

We prospectively enrolled all the patients requiring blood sample collection based on the clinicians’ evaluation who consecutively attended the Pediatric Emergency Department at Sant’Anna e San Sebastiano Hospital in Caserta, Italy, from July 1, 2022, to August 31, 2023. To be enrolled in the study, it was sufficient for the patient to be evaluated in the Pediatric Emergency Department, regardless of the need for admission. Written informed consent was obtained from all participants/parents.

This pediatric ward is located in a general hospital without a Pediatric Intensive Care Unit. Inclusion criteria were age 0–18 years and the need for blood sample collection with serum creatinine measurement, as determined by the pediatric clinical evaluation. Exclusion criteria included the presence of fever or pain at the time of HR measurement, known chronic diseases or anemia, lack of consent to participate in the study, and missing data regarding HR or serum creatinine measurement. Additionally, if there were concerns about the reliability of the measurement due to the child’s anxiety or fear, patients were excluded from the study. The flow chart outlining patients’ enrollment is shown in Supplementary Fig. 1. Our Research Ethical Committee approved the study (approval n°1375/2016).

Due to the study setting, it was challenging to measure the length or height of patients; thus, this data is largely missing and was not included in the analyses or in the calculation of estimated basal creatinine. For this reason, as in our previous studies on patients enrolled in the same setting [5, 8, 9], we used the Hoste(age) equation to estimate basal creatinine [10].

### Data collection

We collected data on age, weight, gender, HR, body temperature, serum creatinine, degree of dehydration, sodium, potassium, chloride, hemoglobin (Hb), hematocrit (Ht), C-reactive protein (CRP), history of chronic diseases, and reasons for attending the Pediatric Emergency Department.

### HR measurement

Heart rate (HR) was measured during observation in the Pediatric Emergency Department before venipuncture, at a time when the child was afebrile and free from any pain that could potentially affect HR. The measurement was taken using the auscultatory method with a stethoscope. The child was positioned as comfortably as possible, even being held in the parent’s arms if necessary, to ensure a calm state. HR was measured by counting beats for 60 s while the child was in the most relaxed state possible. If the child cried or showed signs of fear during the measurement, the HR was reassessed after 5 min and, if needed, again after 10 min. The lowest HR value recorded was used.

### EHRV calculation

As described in our previous study [7], EHRV was calculated using the following formula: [(HR at admission − 50th percentile of HR for age and sex)/HR at admission] × 100. For children aged ≥ 3 years, we used the percentile charts provided by Sarganas et al. [11], and for those younger than 3 years, we used the charts provided by Fleming et al. [12].

### Primary outcome definitions

Our study aimed to prospectively validate the previously determined EHRV cut-off for diagnosing dehydration or AKI [7].

#### Dehydration

Since most of the children in the study did not have recent weight measurements, dehydration was classified as either < 5% or ≥ 5% fluid deficit based on clinical assessment, following the World Health Organization guidelines [1]. Specifically, a child was considered at least moderately dehydrated (≥ 5% fluid loss) if they exhibited two or more of the following signs: irritability, sunken eyes, thirst, or slow skin turgor recovery [1].

#### AKI

For the definition of AKI, we used the estimated basal serum creatinine calculated with previously validated back-calculation methods [10]. The Hoste(age) equation was employed to estimate basal serum creatinine [10], assuming that basal eGFR corresponded to the median age-based eGFR normative values for the children ≤ 2 years of age [10, 13] and eGFR = 120 mL/min/1.73 m^2^ for children > 2 years of age [14].

### Sample size calculation

We determined the required sample size using the Hanley e McNeil method [15], assuming a reference AUROC of 0.70 and a ratio of unaffected to affected patients of 5:1 based on our previous study [7]. We selected a Type I error of 0.05 and Type II error of 0.10 and a null hypothesis value of 0.5. The required sample size was of 156 patients (26 affected patients and 130 unaffected). However, to maximize the statistical power of the study, we decided to increase the number of the enrolled patients.

### Statistical analysis

*P* values < 0.05 were considered statistically significant. Differences in continuous variables were analyzed using the independent-sample *t*-test for normally distributed variables and the Mann–Whitney test for non-normally distributed variables. The normal distribution was assessed by skewness and kurtosis. Values of skewness and kurtosis between − 2 and + 2 were considered indicative of a normal distribution. Qualitative variables were compared by using the chi-square test. Continuous data were presented as mean and standard deviation score (SDS) for normally distributed variables and as median and interquartile range (IQR) for non-normally distributed variables.

EHRV was evaluated as a potential predictor of ≥ 5% dehydration and AKI using receiver-operating characteristic (ROC) curves analysis. We calculated sensitivity, specificity, accuracy, positive and negative likelihood ratio, and positive and negative predictive value (PPV and NPV) of the previously identified EHRV cut-off (24.5%) [7]. Additionally, we calculated the odds ratio (OR) for ≥ 5% dehydration and AKI using logistic regression analysis, adjusting for age and gender, as well as other relevant confounders. In more detail, we adjusted for age, gender, body temperature, and AKI when evaluating the OR for presenting with ≥ 5% dehydration, and for age, gender, body temperature, and ≥ 5% dehydration when evaluating the OR for presenting with AKI. The choice of these parameters was guided by pathophysiological mechanisms potentially influencing HR. Additionally, we corrected for AKI when evaluating the OR for 5% dehydration, and for 5% dehydration when evaluating the OR for AKI to assess the ability of EHRV to independently predict these two conditions. Before conducting these logistic regression analyses, we assessed potential collinearity by calculating the variance inflation factor (VIF) (Supplementary Tables 2 and 3). Variables with a VIF < 2.5—then indicating low collinearity [16]—were included in the model.

All statistical analyses were conducted using SPSS software for Windows. Diagnostic performance calculations were performed using MedCalc.

## Results

We enrolled 256 patients with a mean age of 60.2 ± 48.6 months. The general characteristics of the enrolled population are presented in Supplementary Table 1, while the reasons for accessing the Pediatric Emergency Department are depicted in Supplementary Fig. 2. Among the 256 enrolled patients, 52 had ≥ 5% dehydration, and 50 had AKI. Sixteen patients presented with both ≥ 5% dehydration and AKI.

Patients with ≥ 5% dehydration presented higher HR, EHRV, highest serum creatinine/basal creatinine (HC/BC) ratio, and CRP levels, as well as a higher prevalence of AKI, compared with those without dehydration (Supplementary Table 1). Similarly, patients with AKI exhibited higher EHRV, Ht, and Hb levels, lower serum sodium levels, and a higher prevalence of ≥ 5% dehydration compared to those without AKI (Supplementary Table 1).

ERHV demonstrated significant predictive ability in the ROC curve analysis both for ≥ 5% dehydration (AUROC = 0.71; 95% CI, 0.63–0.78; *p* < 0.001) (Supplementary Fig. 3, panel A) and AKI (AUROC = 0.78; 95% CI, 0.71–0.84; *p* < 0.001) (Supplementary Fig. 3, panel B).

The prognostic accuracy of the previously identified ERHV cut-off of 24.5% for the identification of ≥ 5% dehydration or AKI is shown in Table 1. Multiple logistic regression analysis revealed that patients with EHRV > 24.5% had an OR of 3.5 (95% CI, 1.6–8.0; *p* = 0.003) for presenting with ≥ 5% dehydration, adjusting for age, gender, body temperature, and AKI (Supplementary Table 2). Similarly, the adjusted OR for presenting with AKI, controlling for age, gender, body temperature, and ≥ 5% dehydration, was 3.4 (95% CI, 1.6–7.3; *p* = 0.002) (Supplementary Table 3).

**Table 1:** Prognostic accuracy of the previously identified EHRV cut-off for ≥ 5% dehydration and AKI

Best cut-offs identified at ROC curve analyses	Sensitivity (95% CI)	Specificity (95% CI)	Accuracy (95% CI)	Positive likelihood ratio (95% CI)	Negative likelihood ratio (95% CI)	Positive predictive value (95% CI)	Negative predictive value (95% CI)
EHRV cut-off > 24.5% as predictor of ≥ 5% dehydration	34.0 (21.5–48.3)	83.0 (77.1–88.5)	73.4 (67.6–78.7)	2.1 (1.3–3.4)	0.7 (0.6–0.9)	35.0 (24.9–46.8)	82.9 (79.9–85.6)
EHRV cut-off > 24.5% as predictor of acute kidney injury	36.0 (22.9–50.8)	84.0 (78.2–88.7)	74.6 (68.8–79.8)	2.2 (1.4–3.7)	0.8 (0.6–0.9)	35.3 (25.1–47.1)	84.4 (81.3–87.0)

Abbreviations: *AKI*, acute kidney injury; *EHRV*, estimated percentage of heart rate variation in acute setting compared to the 50th percentile of heart rate according to age and gender; *ROC*, receiver-operating characteristics

## Discussion

The percentage of weight loss is the gold standard for classifying dehydration [1]. Greater weight loss correlates with higher degrees of dehydration and an increased risk of AKI [1]. However, obtaining precise weight loss data is often challenging. In a previous study, we found no significant correlation between parental estimates of weight loss and actual measured weight loss [7]. Additionally, despite the prospective nature of the current study, recent weight measurements were unavailable for most patients. Therefore, identifying reliable and accurate surrogate markers of dehydration is crucial for practical clinical application. In response to this need, we previously assessed the diagnostic performance of EHRV for predicting dehydration and AKI [7]. Notably, a significant relationship was observed between weight loss and EHRV, with an EHRV > 24.5% predicting dehydration and AKI with good accuracy [7]. While EHRV was initially derived from a post hoc analysis of a prospectively enrolled cohort, it had only been validated in a retrospective cohort. This prompted us to validate the utility of EHRV in a different prospective cohort, leading to the design of the current study.

Although an increased HR is a recognized sign of dehydration [1, 17] and has been linked to AKI from a pathophysiological standpoint [18], a literature research using the keywords “[heart rate] AND [dehydration] OR [acute kidney injury]” did not identify any studies investigating the utility of HR in diagnosing dehydration or AKI in children. Except for our previous study [7], no specific HR cut-off for suspecting dehydration or AKI has been established.

However, assessing the renal angina index in the emergency department can provide useful insights for identifying children at risk of developing in-hospital AKI [19] with a better performance compared with EHRV. In fact, the renal angina index had an AUROC of 0.92 (0.86–0.98) for the prediction of inpatient AKI. For AKI prediction, renal angina positive demonstrated a sensitivity of 94% (69–99) and a negative predictive value of 99% (92–100) [19].

In this prospectively enrolled cohort of children, we confirmed the clinical utility of EHRV in predicting dehydration and AKI (Table 1). The increased HR could therefore serve as an early indicator of hemodynamic changes [20], facilitating timely diagnostic testing and intervention [6]. For example, fluid bolus therapy has been associated with a reduction in median HR by 6 beats/min in children presenting to the pediatric emergency department [21].

A limitation of this study—and of EHRV in general—is its applicability only in patients without fever at the time of the HR measurement. Additionally, HR is a highly variable parameter that can be influenced by fear or anxiety. In this study, we took care to create an environment conducive to unbiased HR measurement, though we acknowledge that this may not always be feasible in routine clinical practice. Moreover, while the literature suggests that the auscultatory method (apex listening) is reasonably accurate when compared to electrocardiography [22], a median discrepancy of 8 beats/min is described in infants [22]. Additionally, a systematic review revealed that heart rates measured using automated techniques (e.g., electrocardiography) were significantly higher than those measured manually [12]. Consequently, in our cohort, particularly among infants, we may have underestimated HR, potentially leading to an underestimation of 5% dehydration and/or AKI. Specifically, considering an underestimation of 8 beats/min for infants [22], the EHRV could be underestimated by approximately − 5.7% at 3 months of age and − 6.8% at 24 months of age. However, since Fleming et al. [12] derived HR percentiles using several studies that employed different HR measurement methods, including 10,228 manual measurements from 6 studies, the fact that we used their percentiles [12] for younger children may have partially mitigated this limitation.

Another limitation is that AKI was diagnosed based on calculated basal creatinine. However, this method has been validated in the literature [10, 23]. Moreover, we only had a single serum creatinine measurement for the enrolled patients, which could have been influenced by the patients’ blood volume. It is possible that patients diagnosed with AKI may actually have only functional AKI rather than a more severe intrinsic form. Nevertheless, identifying these patients remains crucial to preventing progression to intrinsic AKI with tubular damage.

Finally, we did not collect data about potential patient admissions or biochemical exams during the hospital stay. This could have been valuable for evaluating the evolution of AKI following adequate rehydration.

In conclusion, our study prospectively validates that an EHRV greater than 24.5% could serve as a marker for suspecting dehydration or AKI. Our findings have been validated in a Pediatric Emergency Department and could potentially be applied to initial evaluations of children in other settings as well. However, further validation across diverse patient populations and clinical settings is warranted. While HR monitoring is a valuable clinical parameter that can provide early indications of hemodynamic instability and potential dehydration or AKI, its non-specific nature and variability among individuals limit its use as a standalone diagnostic tool. Its primary utility lies in serving as an early warning sign, prompting further diagnostic evaluation and monitoring the effectiveness of therapeutic interventions.

## Supplementary Information

Below is the link to the electronic supplementary material.

Supplemental Table 1 (DOC 48 kb)

Supplemental Table 2 (DOCX 14 kb)

Supplemental Table 3 (DOCX 14 kb)

Supplemental Fig. 1 Flow chart describing patients’ enrollment (PNG 202 kb)

High resolution image (TIF 4267 kb)

Supplemental Fig. 2 Reasons for access to the Pediatric Emergency Department. Among other causes are included: 1 patient with Bell’s palsy, 1 patient with Brief Resolved Unexplained Event (BRUE), 2 patients with diabetic ketoacidosis, 1 patient with epistaxis, 2 patients with jaundice, 2 patients with skin rash, 1 patients with stomatitis, and 3 patients with suspect of intoxication. (PNG 410 kb)

High resolution image (TIFF 1194 kb)

Supplemental Fig. 3 ROC Curve analyses of EHRV. Panel A: ROC curve for ≥5% dehydration. Panel B: ROC curve for AKI. (PNG 149 kb)

High resolution image (TIF 470 kb)

## Data Availability

The datasets generated during and/or analysed during the current study are available from the corresponding author on request.

## Abbreviations

AKI: Acute kidney injury
AUROC: Area under ROC curve
CKD: Chronic kidney disease
EHRV: Estimated percentage of heart rate variation in acute setting compared with 50th percentile of HR according to age and gender
HR: Heart rate
NPV: Negative predictive value
OR: Odds ratio
PPV: Positive predictive value
ROC: Receiver-operating characteristics
VIF: Variance inflation factor

## Code availability

N/A.
