# Supplementary material for: Heart rate to identify non-febrile children with dehydration and acute kidney injury in emergency department: a prospective validation study
Source: Eur J Pediatr. 2024 Sep 16;183(11):5043–8. doi: 10.1007/s00431-024-05770-6 (PMC11473630; doi:10.1007/s00431-024-05770-6)
Supplement: Supplementary file 1 — (DOC 48 kb) [file 431_2024_5770_MOESM1_ESM.doc]

**Supplementary Table 1. Main clinical, demographic, and biochemical characteristics of the enrolled patients, comparing those with and without 5% dehydration and those with and without AKI.**

|  | **All patients**  **No.=256** | **<5% dehydration**  **No.= 204** | **≥5% dehydration**  **No.= 52** | **p** | **AKI (no)**  **No.= 206** | **AKI (yes)**  **No.= 50** | **p** |
| --- | --- | --- | --- | --- | --- | --- | --- |
| **Age, yr, mean (SDS)** | 60.2 (48.6) | 63.0 (50.4) | 51.4 (42.1) | 0.11 | 62.2 (51.1) | 53.9 (40.3) | 0.29 |
| **Male sex, No. (%)** | 141 (55.1) | 106 (51.9) | 35 (67.3) | 0.05 | 116 (56.3) | 25 (50.0) | 0.42 |
| **HR, beats/min, mean (SDS)** | 119.6 (22.5) | 116.8 (22.3) | 130.6 (19.6) | <0.001 | 118.5 (21.7) | 124.1 (25.5) | 0.12 |
| **EHRV, mean (SDS)** | 13.1 (16.8) | 11.1 (16.7) | 21.7 (14.5) | <0.001 | 10.1 (16.7) | 25.6 (10.8) | <0.001 |
| **Creatinine, mg/dL, mean (SDS)** | 0.40 (0.13) | 0.39 (0.12) | 0.42 (0.16) | 0.28 | 0.37 (0.11) | 0.54 (0.13) | <0.001 |
| **HC/BC ratio, mean (SDS)** | 1.2 (0.38) | 1.1 (0.34) | 1.3 (0.50) | 0.02 | 1.0 (0.23) | 1.7 (0.32) | <0.001 |
| **AKI, No. (%)** | 50 (19.5) | 34 (16.7) | 16 (30.8) | 0.02 | N/A | N/A | N/A |
| **≥5% dehydration, No. (%)** | 52 (20.3) | N/A | N/A | N/A | 36 (17.5) | 16 (32.0) | 0.02 |
| **Serum sodium level, mEq/L,**  **mean (SDS)** | 136.9 (3.1) | 137.0 (3.0) | 136.3 (3.6) | 0.1 | 137.1 (3.1) | 136.1 (3.2) | 0.04 |
| **Serum chloride levels, mEq/L,**  **mean (SDS)** | 101.2 (3.0) | 101.0 (2.8) | 101.6 (3.6) | 0.2 | 101.0 (2.8) | 102.0 (3.5) | 0.06 |
| **Serum potassium levels, mEq/L, mean (SDS)** | 4.4 (0.57) | 4.4 (0.6) | 4.4 (0.58) | 0.68 | 4.4 (0.58) | 4.3 (0.53) | 0.06 |
| **Haematocrit, %, median (IQR)** | 36.0 (4.7) | 36 (4.3) | 36.7 (6.2) | 0.71 | 36 (4.6) | 37.5 (4.7) | 0.007 |
| **Haemoglobin, g/dL, median (IQR)** | 12.3 (1.9) | 12.3 (1.8) | 12.3 (2.1) | 0.78 | 12.1 (1.9) | 12.6 (1.5) | 0.008 |
| **C-RP, mg/dL, median (IQR)** | 0.50 (2.9) | 0.49 (2.9) | 0.87 (3.2) | 0.02 | 0.52 (2.9) | 0.58 (3.2) | 0.90 |

Mean and SDS are shown for normally distributed variables while median and IQR are shown in case of non-normality.

*Abbreviations:* AKI, acute kidney injury; C-RP, C-reactive protein; EHRV, estimated heart rate variation in acute setting in comparison with 50^th^percentile of heart rate; HR, hearth rate; HC/BC, highest serum creatinine/basal creatinine; IQR, interquartile range; SDS, standard deviation score.
