# Supplementary material for: Heart rate to identify non-febrile children with dehydration and acute kidney injury in emergency department: a prospective validation study
Source: Eur J Pediatr. 2024 Sep 16;183(11):5043–8. doi: 10.1007/s00431-024-05770-6 (PMC11473630; doi:10.1007/s00431-024-05770-6)
Supplement: Supplementary file 2 — (DOCX 14 kb) [file 431_2024_5770_MOESM2_ESM.docx]

**Supplementary Table 2. Multiple logistic regression analysis for presenting with ≥5% dehydration.**

| **Variables** | **OR** | **95%CI** | **p** | **VIF** |
| --- | --- | --- | --- | --- |
| **EHRV>24.5%** | 3.5 | 1.6-8.0 | 0.003 | 1.150 |
| **Age^a^, months** | 0.98 | 0.97-0.99 | 0.03 | 1.093 |
| **Female gender** | 0.47 | 0.24-0.93 | 0.03 | 1.012 |
| **Body temperature^b^, °C** | 1.3 | 0.89-2.0 | 0.16 | 1.034 |
| **AKI** | 1.5 | 0.67-3.2 | 0.31 | 1.058 |

^a^1 month increase in age

^b^1 °C degree increase in body temperature

*Abbreviations:* AKI, acute kidney injury; CI, confidence interval; EHRV, estimated heart rate variation in acute setting in comparison with 50^th^percentile of heart rate; OR, odds ratio; VIF, **Variance Inflation Factor.**
