# Supplementary figures and images for: Heart rate to identify non-febrile children with dehydration and acute kidney injury in emergency department: a prospective validation study
Source: Eur J Pediatr. 2024 Sep 16;183(11):5043–8. doi: 10.1007/s00431-024-05770-6 (PMC11473630; doi:10.1007/s00431-024-05770-6)

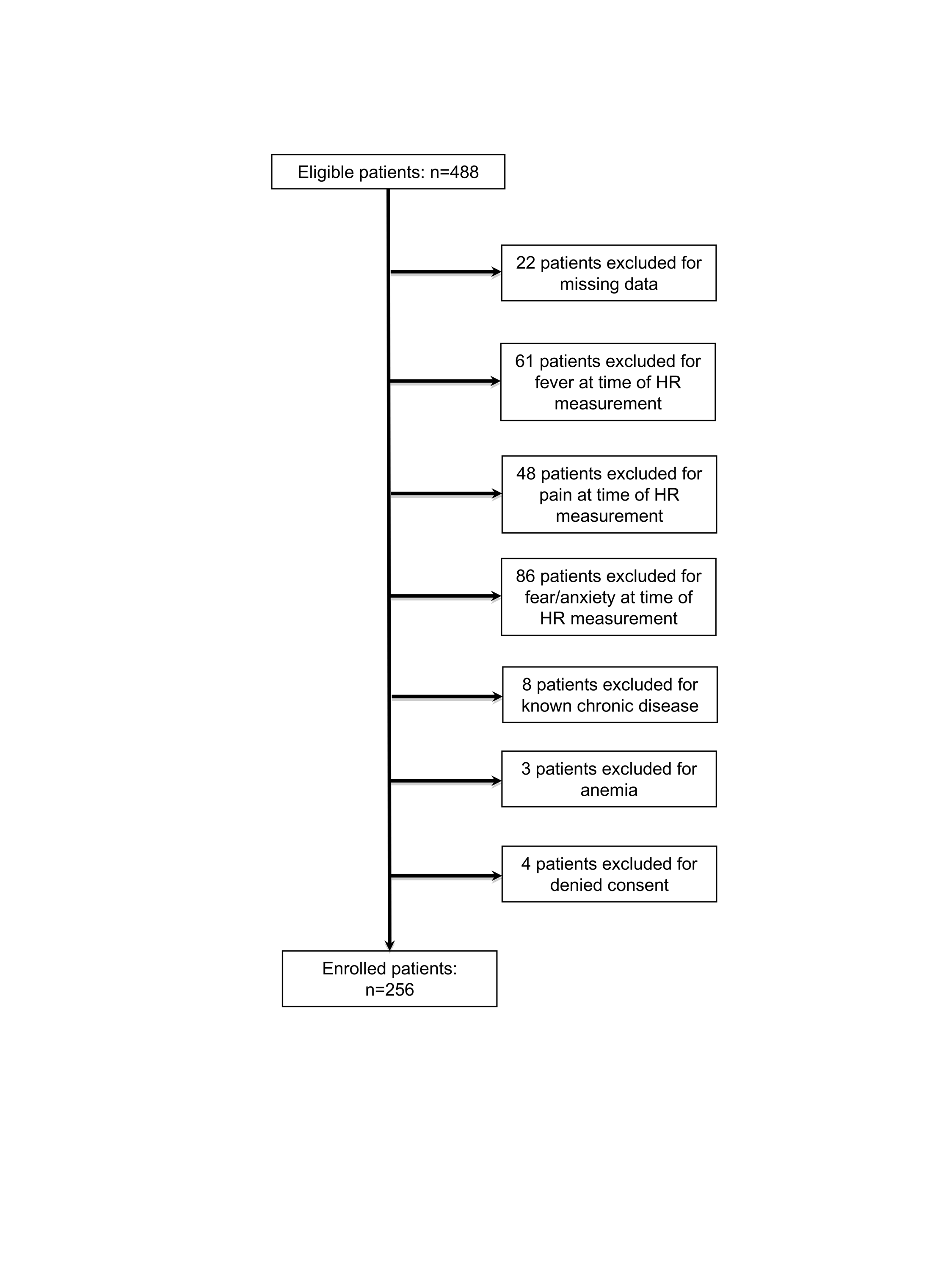

Supplement: Supplementary file 4 — Flow chart describing patients’ enrollment (PNG 202 kb) [file 431_2024_5770_Fig1_ESM.png]

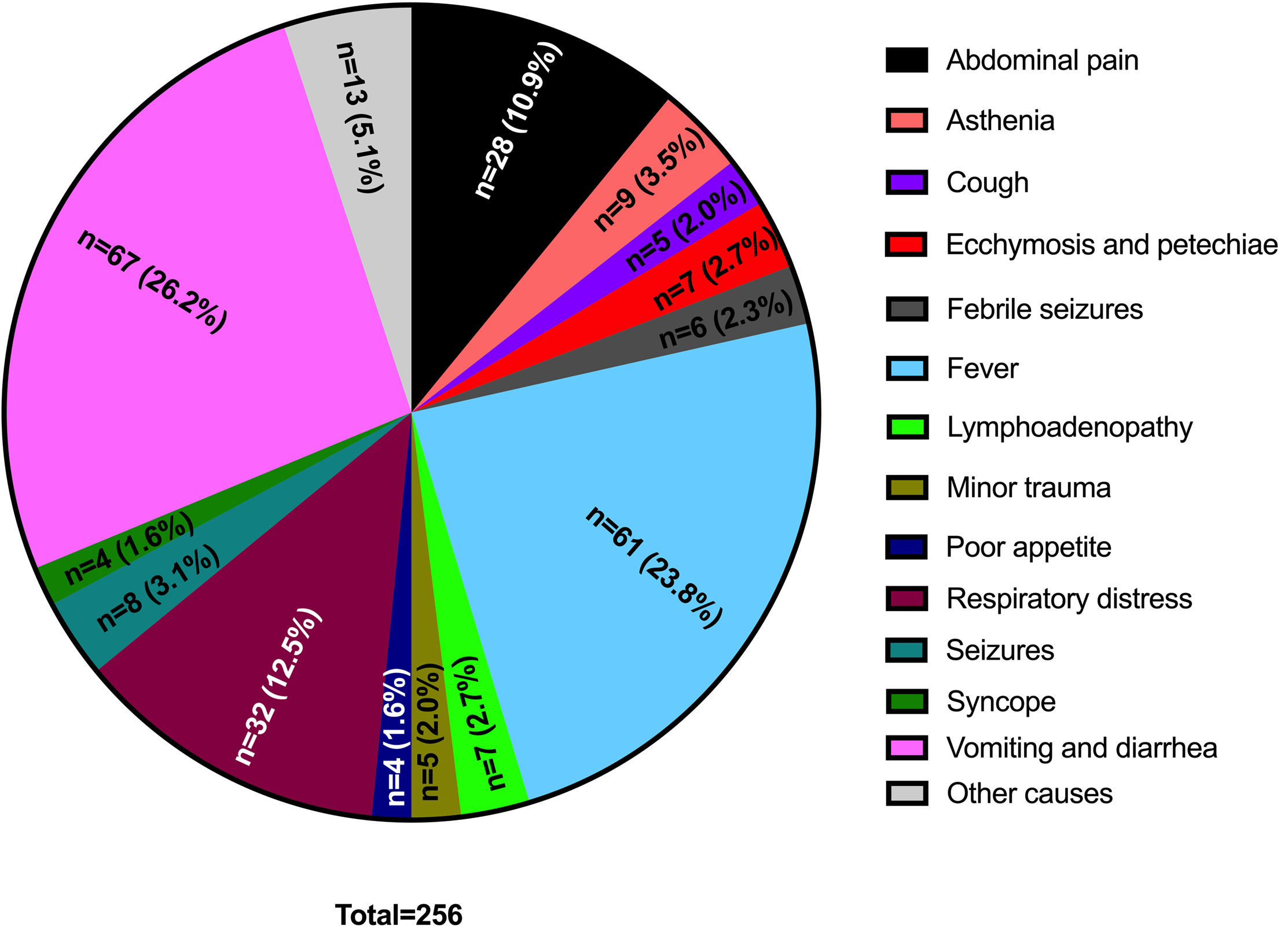

Supplement: Supplementary file 6 — Reasons for access to the Pediatric Emergency Department. Among other causes are included: 1 patient with Bell’s palsy, 1 patient with Brief Resolved Unexplained Event (BRUE), 2 patients with diabetic ketoacidosis, 1 patient with epistaxis, 2 patients with jaundice, 2 patients with skin rash, 1 patients with stomatitis, and 3 patients with suspect of intoxication. (PNG 410 kb) [file 431_2024_5770_Fig2_ESM.png]

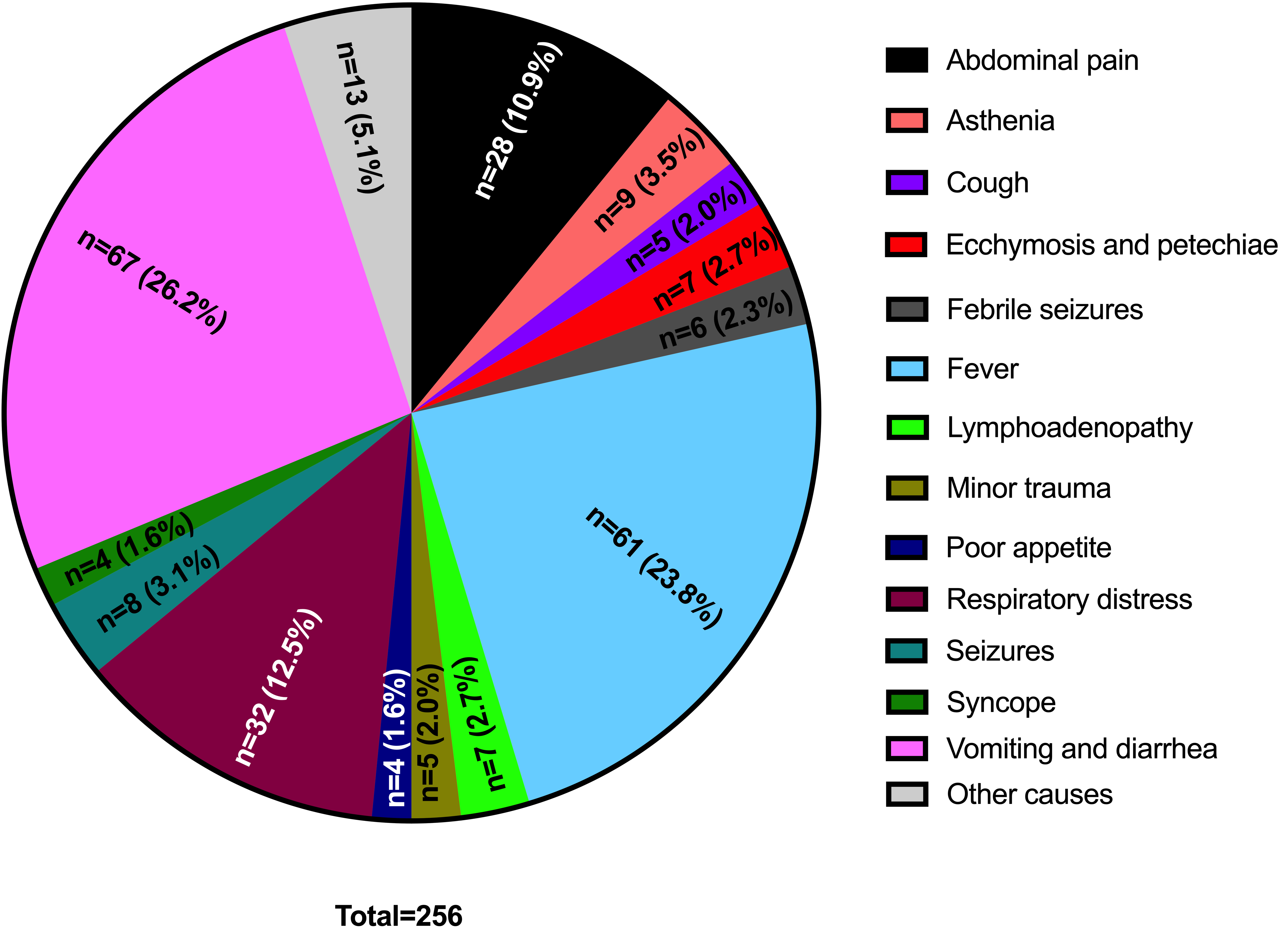

Supplement: Supplementary file 7 — High resolution image (TIFF 1194 kb) [file 431_2024_5770_MOESM5_ESM.tiff]

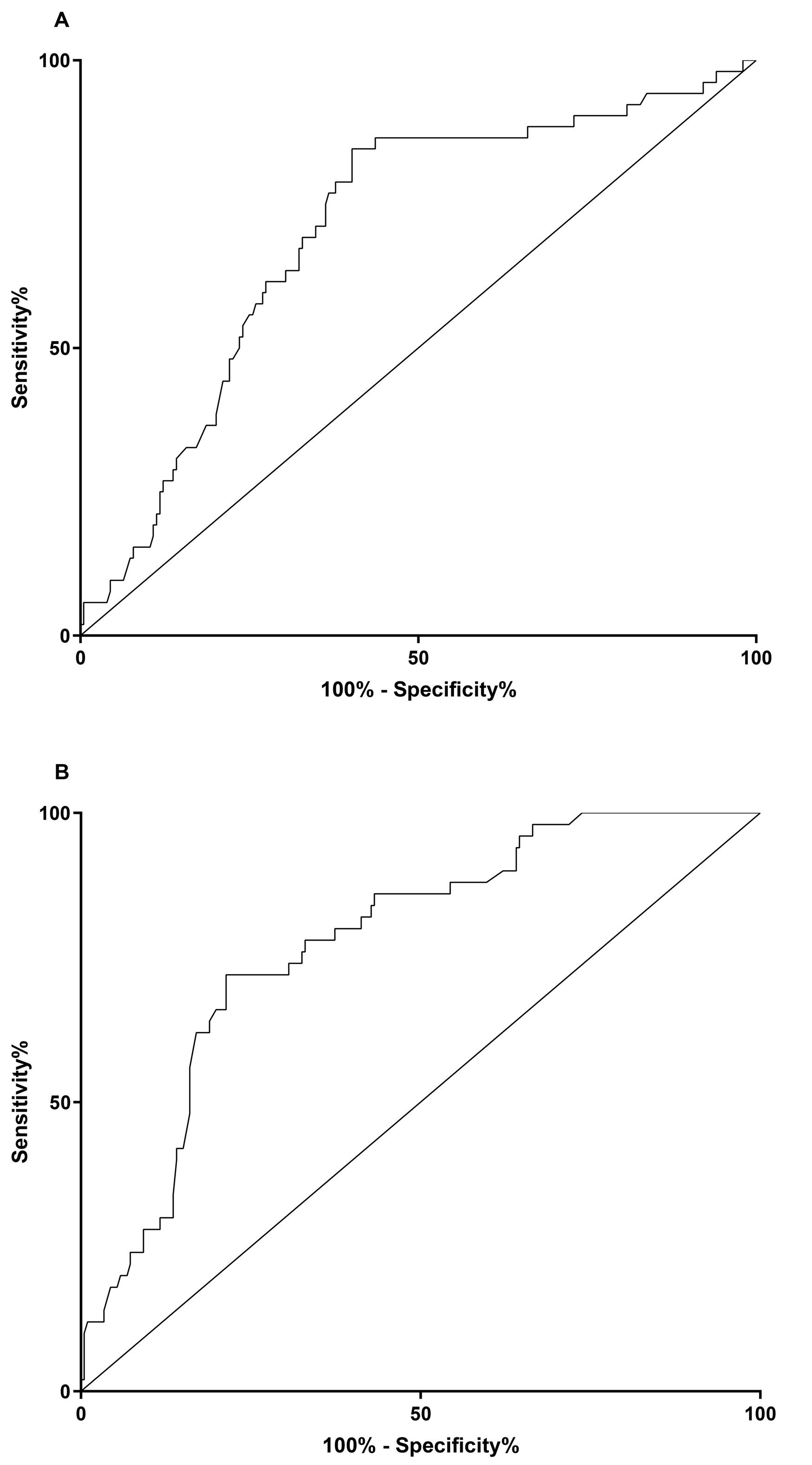

Supplement: Supplementary file 8 — ROC Curve analyses of EHRV. Panel A: ROC curve for ≥5% dehydration. Panel B: ROC curve for AKI. (PNG 149 kb) [file 431_2024_5770_Fig3_ESM.png]

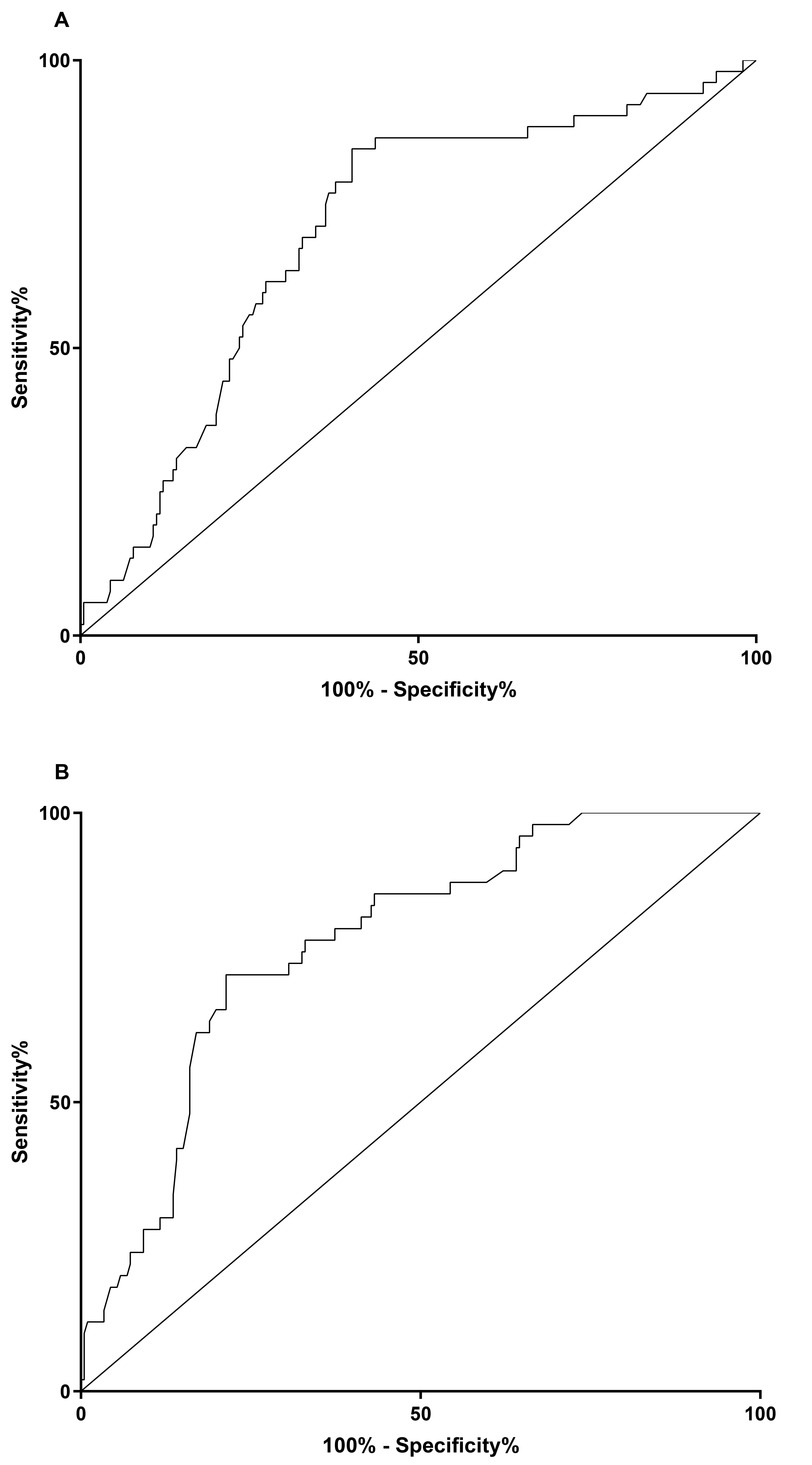

Supplement: Supplementary file 9 — High resolution image (TIF 470 kb) [file 431_2024_5770_MOESM6_ESM.tif]
